# Molecular phenotyping of laboratory mouse strains using 500 multiple reaction monitoring mass spectrometry plasma assays

**DOI:** 10.1038/s42003-018-0087-6

**Published:** 2018-06-27

**Authors:** Sarah A. Michaud, Nicholas J. Sinclair, Helena Pětrošová, Andrea L. Palmer, Adam J. Pistawka, Suping Zhang, Darryl B. Hardie, Yassene Mohammed, Azad Eshghi, Vincent R. Richard, Albert Sickmann, Christoph H. Borchers

**Affiliations:** 1 University of Victoria - Genome British Columbia Proteomics Centre, Vancouver Island Technology Park, #3101- 4464, Markham St., Victoria, BC V8Z 7X8 Canada; 2 Center for Proteomics and Metabolomics, Leiden University Medical Center, Albinusdreef 2, 2333 ZA Leiden, The Netherlands; 3 Jewish General Hospital Proteomics Laboratory, McGill University, Lady Davis Institute, 3755 Chemin de la Côte-Sainte-Catherine, Montréal, QC H3T 1E2 Canada; 4 Leibniz-Institut für Analytische Wissenschaften-ISAS-e.V, Dortmund, 44139 Germany; 5 Department of Biochemistry and Microbiology, University of Victoria, Petch Building Room 207, 3800 Finnerty Rd., Victoria, BC V8P 5C2 Canada; 6 Department of Oncology, Jewish General Hospital Proteomics Centre, McGill University, 3755 Cote-Ste-Catherine Road, Montreal, QC H3T 1E2 Canada

## Abstract

Mouse is the predominant experimental model for the study of human disease due, in part, to phylogenetic relationship, ease of breeding, and the availability of molecular tools for genetic manipulation. Advances in genome-editing methodologies, such as CRISPR-Cas9, enable the rapid production of new transgenic mouse strains, necessitating complementary high-throughput and systematic phenotyping technologies. In contrast to traditional protein phenotyping techniques, multiple reaction monitoring (MRM) mass spectrometry can be highly multiplexed without forgoing specificity or quantitative precision. Here we present MRM assays for the quantitation of 500 proteins and subsequently determine reference concentration values for plasma proteins across five laboratory mouse strains that are typically used in biomedical research, revealing inter-strain and intra-strain phenotypic differences. These 500 MRM assays will have a broad range of research applications including high-throughput phenotypic validation of novel transgenic mice, identification of candidate biomarkers, and general research applications requiring multiplexed and precise protein quantification.

## Abstract (web summary)

Christoph Borchers, Albert Sickmann and colleagues present multiple reaction monitoring/mass spectrometry assays for reproducible quantification of 500 plasma proteins in mice. They report reference protein concentrations in five mouse strains, and inter- and intra-strain phenotypic variations.

## Introduction

Research findings from mouse models have contributed to our understanding of the underlying mechanisms of human pathologies, and are important for development and testing of novel diagnostic tools and treatment strategies^1–3^. Advances in genome manipulation techniques now allow rapid development of mouse strains with specific genotypes, which mimic hundreds of human diseases and conditions^4, 5^. However, the detailed characterization and validation of these models remain challenging, due to the limited number of tools that enable reliable and high-throughput molecular phenotyping.

Current high throughput strategies for molecular phenotyping rely on gene expression screening methodologies, such as quantitative real-time PCR, RNA-Seq, or microarray techniques^6, 7^. Measured differences on the mRNA level, however, do not necessarily equate to protein abundance, and may therefore be an inaccurate assessment of phenotype^8–10^. Protein expression profiling is routinely performed using affinity-based assays, such as immunoblot and enzyme-linked immunosorbent assay (ELISA), which have a limited potential for multiplexing, and are dependent on availability and quality of protein-specific antibodies^11, 12^. As a result, protein profiling studies often focus on a narrow range of proteins for which affinity-based assays already exist^13^. Quantitative mass-spectrometric techniques offer an alternative approach for multiplexed proteome profiling without the need of specific antibodies or probes^14–16^.

For protein quantification, multiple reaction monitoring (MRM) tandem mass spectrometry (MS/MS) coupled with stable isotope-labelled internal standard peptides is unmatched in precision and specificity^17^. In the present study, MRM was used to develop quantitative assays for molecular phenotyping in mouse blood plasma. Plasma is a dynamic fluid that reflects physiological and pathological states of the organism, and is routinely used to monitor acute events such as disease progression and reoccurrence, and treatment efficacy in humans^18–20^. Plasma proteins are therefore ideal targets for characterization of mouse models and these proteins can be specifically and precisely quantified in high throughput, via MRM. Using MRM, hundreds of preselected peptides and inferred proteins can be monitored in plasma, with excellent intra-laboratory and inter-laboratory reproducibility^17, 21^. The broad dynamic range (10^4^–10^6^) of MRM allows reproducible measurement of proteins with concentrations as low as 2–10 ng mL^−1^ in non-depleted and un-fractioned plasma^22^, providing an innate representation of the plasma proteome. Moreover, multiplexed MRM experiments can be executed on needle prick volumes of blood to monitor >200 surrogate peptides in a single liquid chromatography (LC) injection^21^ using only a fraction of the sample volume. Measurement of plasma protein abundance can therefore be performed repeatedly for precision and extended to include more targets for increased throughput.

In contrast to well characterized affinity based assays which are available from various vendors, precise quantitative MRM assays have yet to be developed and made available to the broader researcher community. To support scientists in developing high-quality MRM assays in experimental workflows, the Clinical Proteomic Tumour Analysis Consortium (CPTAC) proposed guidelines for MRM assay development and validation^23, 24^. In strict accordance with the CPTAC document^25^, 500 highly sensitive and precise MRM assays were developed, targeting 500 proteins in mouse plasma, covering approximately 20% of the predicted mouse plasma proteome^26^, or ~15% if the human plasma proteome is used as a reference^27^. Unique linear standard curves spanning a concentration range of three orders of magnitude were designed for each endogenous peptide target in plasma, using combinations of synthetic homologous peptides composed of either natural (^12^C/^14^N) or stable heavy (^13^C/^15^N) isotope (SIS) amino acids^25^. The broad applicability of these assays was subsequently demonstrated by quantification of reference protein concentrations in common laboratory mouse strains.

## Results

### Protein and peptide selection

Protein targets for MRM assays were selected based on discovery experiments using LC-MS/MS in the data-dependent mode, which identified 297 plasma proteins in non-depleted, unfractioned plasma from C57BL/6 mice obtained from BioReclamation (C57BL/6/BR). Additional protein targets were selected based on clinical and biological relevance, as evidenced by original research articles^26, 28–34^. PeptidePicker software^35^ was then used to select 1663 MRM-compatible surrogate peptides for synthesis. Peptides displaying satisfactory yield and that were soluble in compatible solvent were subsequently screened for their suitability for performing quantitative MRM, taking into consideration factors such as specificity (interference) and reproducibility. A total of 500 peptides were subsequently selected for quantitative MRM assays.

### MRM assay development

**Fig. 1:**
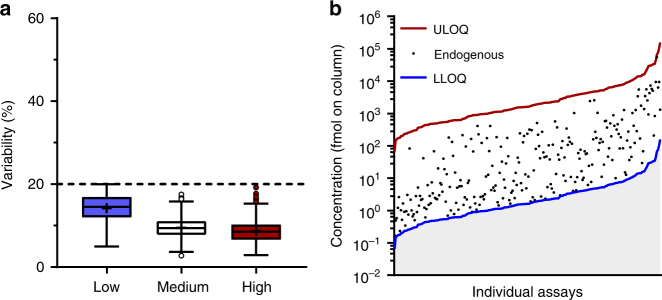
Repeatability and dynamic range of designed assays (CPTAC Experiment 2). **a** Total variability of peak areas, as calculated from the intra-assay and inter-assay CoVs across five independent experiments. Box and whisker plots were generated using the Tukey method; mean (+) and outliers (circles) are shown. Blue, white, and red boxes correspond to the total variability at low (2.5× or 5× assay LLOQ), medium (50× assay LLOQ) and high (500× assay LLOQ) concentrations of the spiked-in SIS peptide, respectively. The dotted line represents the 20% cut-off, as defined by CPTAC. **b** The dynamic range of the designed assays. Assay LLOQ (blue line), assay ULOQ (red line), and the detectable concentrations of the respective endogenous analyte (black dots, *N* = 367) in pooled plasma of C57BL/6 mice (*N* = 30) are shown

The lower limits of quantification (LLOQs) for each endogenous target peptide were determined in accordance with CPTAC experiment 1 (Supplementary Fig. 1), and ranged from 0.03 to 157.74 fmol injected onto the LC column (Supplementary Data 1 and Supplementary Data 2). To measure the variation in MRM assay performance, SIS peptides were spiked in at 2.5 × or 5 × LLOQ (in parallel), 50 × LLOQ and 500 × LLOQ, corresponding to low, medium, and high quality control concentrations and the coefficients of variation determined at each concentration. According to the CPTAC guidelines the coefficients of variation (CoVs) were required to be <20% in samples spiked with high-SIS and medium-SIS concentrations, and with at least one of the two lowest (2.5 × or 5 × LLOQ) SIS concentrations. All assays met this criterion (Fig. 1a). The lowest point on the standard curve for each validated assay was defined as the assay LLOQ if the CoV at 2.5 × LLOQ was <20% (Fig. 1b). For assays having a CoV > 20% at 2.5 × LLOQ, the assay LLOQ was changed to 2 × LLOQ, and assays with a CoV > 20% at 5 × LLOQ were not included in the panel of 500 assays.

**Fig. 2:**
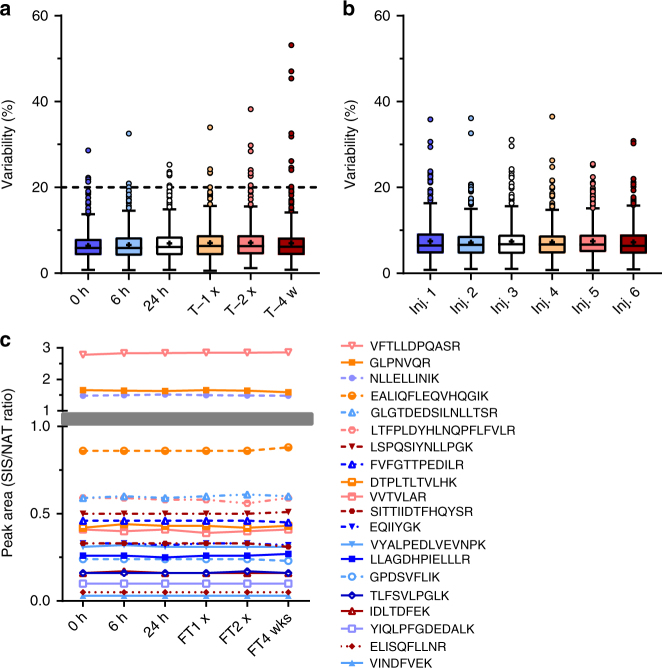
Stability of designed assays (CPTAC Experiment 4). **a** Intra-assay variability in SIS/NAT peak area ratio. Triplicate aliquots of the samples were analyzed by duplicate LC/MRM-mass spectrometry injections after 0, 6, or 24 h on the autosampler (4 °C). Additional aliquots were frozen at −80 °C, and analyzed identically after thawing (T−1×), after a second freeze-thaw cycle (T−2×), or after 4 weeks at −80 °C (T−4w). **b** Inter-assay variability in SIS/NAT peak area ratios comparing each of the six injections across all time points and freeze-thaw cycles. Box and whisker plots were generated using the Tukey method; mean (+) and outliers (circles) are shown. The dotted line represents the 20% cut-off, as defined by CPTAC. **c** SIS/NAT peak area ratios of 20 representative peptides across six experimental time points. T, freeze-thaw; w, weeks; Inj, injection

In order to determine the stability of the peptides in the samples, pooled plasma samples that had been digested and spiked with SIS peptides were kept for various periods of time under different storage conditions and temperatures, before subsequent analysis by MRM. Triplicate aliquots of digested mouse plasma samples were spiked with SIS peptides at a concentration in the mid-range of the assay, along with a constant concentration (200 fmol for each injection) of the corresponding natural (unlabelled) peptides (NAT) (Table 1). Samples were analyzed immediately (0 h), and after 6 or 24 h storage at 4 °C. Aliquots of the samples were also frozen at -80 °C, and analyzed after thawing (T−1×), after a second freeze-thaw cycle (T−2×), or after 4 weeks in the freezer (T−4w) (Fig. 2a). The SIS/NAT signal ratios were measured at each time point and storage temperature, and the CoVs compared across the six experimental conditions (Fig. 2b), as well as across replicates within the same time point/freeze-thaw cycle (Fig. 2a). In total, 96.6% (483/500) of the assays remained stable (CoV < 20%) under the conditions defined in the CPTAC guidelines (Supplementary Figs. 1, 2, and Supplementary Data 3), and 20 representative peptides are displayed in Fig. 2c exhibiting stability over time and after multiple freeze-thaw cycles.

**Table 1:** Overview of validation and quantitation experiments

CPTAC experiment	Outcome	Plasma sample^a^	Standard (fmol on LC column for each injection)^b^	Normalizer (fmol on LC column for each injection)^b^	Experimental design^c^
1	Response curve	Pooled (*N* = 30)^d^	SIS 20,000 to 0.16; dilution pattern: 1:10:10:5:2:2:2:2:2:2:2:2	NAT 200	Digested sample was spiked with serial dilutions of SIS peptides and analyzed on the same day
2	Validation of assay repeatability	Pooled (*N* = 30)^d^	SIS 2.5× and 5× (low), 50× (medium), and 500× (high) assay LLOQ	NAT 200	Five aliquots of the same digested sample were spiked and analyzed on five different days
3	Assessment of assay selectivity	Individual (*N* = 6)^d^	NAT no spike, 50×, and 500× assay LLOQ	SIS 100× assay LLOQ	Six biological replicates of the digested matrix were spiked and analyzed on the same day.
4	Validation of assay stability	Pooled (*N* = 30)^d^	SIS 100× assay LLOQ	NAT 200	Six aliquots of the digested and spiked sample were analyzed after being stored at 4 and −80 °C for various periods of time
5	Reproducible detection of the endogenous analyte	Pooled (*N* = 30^d^	–	SIS 100× assay LLOQ	Five aliquots of the same sample were digested, spiked, and analyzed on five different days
Quantitation	Reference range of target protein concentrations	Individual (*N* = 6 for each strain)^e^	–	SIS 100× assay LLOQ	Individual samples were digested, spiked, and analyzed. Concentration ranges of endogenous analytes were determined using a calibration curve prepared in a surrogate matrix

*SIS* stable isotope labelled peptide, *NAT* natural (unlabelled) peptide, *LLOQ* lower limit of quantification

^a^*N* refers to a number of biological replicates

^b^In all experiments except for the CPTAC Experiment 1, concentration-balanced mixtures of standards were used in order to reflect the dynamic range of the individual assays and the concentrations of the respective endogenous analytes in mouse plasma. Validated assay LLOQ values for each assay are listed in Supplementary Data 2

^c^Samples were analyzed using a single injection (Quantification), two injections (Experiment 4) or three injections (Experiments 1, 2, 3, and 5). According to the CPTAC guidelines, different days were defined as different calendar days with digestions separated by at least 16 h

^d^Plasma samples from C57BL/6 mice (BioReclamationIVT)

^e^Plasma samples from C57BL/6 mice (BioReclamationIVT), CD1, 129S1/SvlmJ, NOD/SCID/J#1303, Balb/cJ, C57BL/6/CRL, and C57BL/6J (Toronto Centre for Phenogenomics)

To determine the reproducibility of detection of an endogenous analyte, five aliquots of the same pooled mouse plasma sample were digested, spiked with SIS peptides, and analyzed by MRM (Supplementary Fig. 1 and Table 1). This process was repeated on five different days, and intra-assay, inter-assay, and the total variability was calculated as described in Experiment 2 for all of the twenty-five samples. For 252 (50.4%) assays, the total variability was less than 20%, demonstrating that these endogenous peptides can be reproducibly detected in normal mouse plasma by MRM (Supplementary Data 4). It is important to note that reproducibility in Experiment 5 is affected by the concentration of the endogenous peptide in the plasma matrix, with low-abundance peptides displaying relatively higher CoVs, as expected. For the remaining 248 peptides, 115 (23.0%) endogenous peptides were below the LLOQ in pooled plasma of C57BL/6 mice (i.e., CoV > 20 %) and 133 (26.6%) analytes, the endogenous analyte in the pooled plasma from C57BL/6BR mice were below the LLODs. However, 41 of these 248 analytes were quantifiable via 2D-LC/MS using single-point measurement (Supplementary Data 4). Assays corresponding to these targets may find applications in other laboratory mouse strains and in mouse models of disease, where the target protein concentrations in plasma might be elevated.

To determine the specificity of the designed assays, the CPTAC guidelines recommend analyzing the spiked-in peptide responses in six biological replicates of the matrix, in this case plasma samples collected from six individual C57BL/6BR mice. The slope of the response line measured in each sample must lie within 10% of the mean of all six biological replicates to satisfy the CPTAC guidelines for selectivity (see Supplementary Fig. 1 Experiment 3). Plasma samples collected from individual mice contain varying unknown concentrations of the endogenous analyte, preventing the use of the respective NAT peptide as the normalizer. This limitation was overcome by the use of a forward response curve.

In the forward response curve, plasma samples were spiked with three concentrations of each NAT peptide reflecting the dynamic range of the assay, along with a constant concentration of the corresponding SIS peptides (Supplementary Fig. 1 and Table 1). Samples were analyzed by MRM, and the resulting NAT/SIS signal ratios were used to calculate the slope of the response line for each sample, and the mean slope of the response line for each assay. For 278 of the 498 assays (55.8%), the slope of the response line in all individual samples lay within 10% of the mean (Supplementary Data 5 and Supplementary Fig. 1).

In summary, the analytical performance of the MRM assays was validated in accordance with the CPTAC guidelines^24, 25^. More than half of the assays (55.6%) displayed individual slope within 10% of the mean, which passes selectivity criteria as designated in CPTAC experiment 3. All 500 assays were reproducible across repeated experiments and technical replicates, and the majority (96.6%) of samples were stable after varying storage conditions and after multiple freeze thaw cycles (<20% CoV). Furthermore, reproducible quantification of endogenous peptides was validated for 252 assays (*N* = 5 per day, on 5 different days (<20% CoV; CPTAC experiment 5), on pooled plasma of C57BL/6BR mice. Assays which had CoVs of >20% or where the endogenous concentration was below the LLOQ may still be useful for studies of disease models, where the endogenous peptide is present in plasma at higher concentrations, or under experimental conditions where the differences in protein abundance are 2–3 standard deviations above the CoV. Additionally, these assays may be useful when platforms with enhanced sensitivity are used, as exemplified in the present study by online two dimensional high-pH—low-pH reversed-phase-reversed-phase (2D RPRP) LC-MRM^36^ (Supplementary Data 4).

### Quantification of proteins in plasma from five mouse strains

**Fig. 3:**
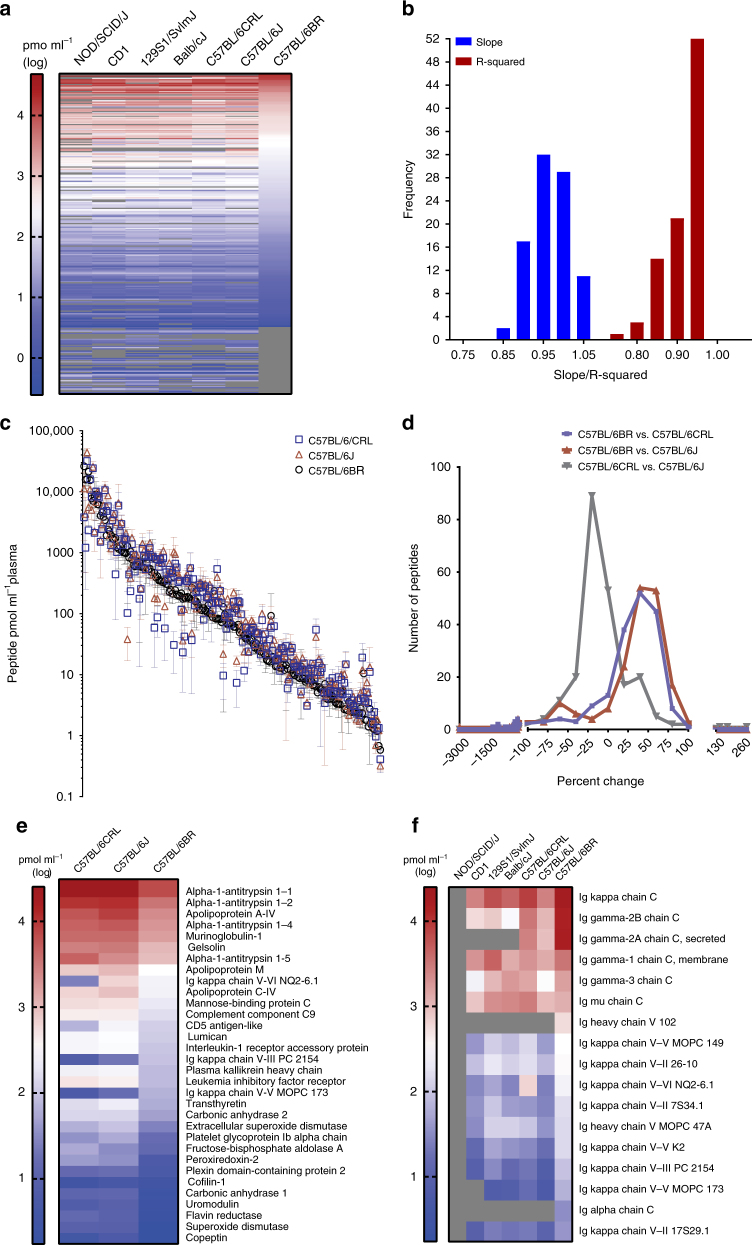
Plasma protein profiles of five laboratory mouse strains. **a** Heat map presenting plasma protein abundances (log-transformed) in each mouse strain (male and female protein abundance were combined). **b** Frequency distribution of the slope and *R*^2^ values calculated from 91 distinct linear regression analyses of plasma protein abundance (log transformed) across all male and female mouse strains and colonies (5 strains, 7 colonies, male and female separated, resulting in 91 distinct linear regression curves). **c** Intra-strain comparison of the abundance of plasma proteins in C57BL/6 mice obtained from three different vendors (*y* axis is in antilog scale). **d** Frequency distribution of percent differences in proteins in C57BL/6 mice from three different vendors. All differences in peptide abundance are shown, including those without statistical significance. **e** Intra-strain variability of protein abundance in plasma of C57BL/6 mice. Only proteins whose abundances differed between at least two C57BL/6 strains are shown (*p* < 0.05 by two-way ANOVA with Tukey’s correction for multiple comparisons). **f** Strain-specific abundance of immunoglobulins (Ig) in plasma. Abundances are shown only for target analytes detected within the dynamic range of the respective assays in plasma from at least four individual mice for each mouse strain; grey coloring indicates analytes that were not detected or were not quantifiable in plasma of the respective mouse strains. *N* = six mice/strain (three male and three female). Means of log-transformed data are shown; concentrations are those of the surrogate peptides (pmol mL^−1^ of plasma). CRL, Charles River Laboratories; J, Jackson Laboratory; and BR, BioReclamationIVT

The panel of 500 MRM assays was used to determine the concentrations of the target proteins in plasma from five commonly-used laboratory mouse strains: C57BL/6, BALB/c, 129S1, CD1 and NOD/SCID (Fig. 3a; Table 2, Supplementary Data 6, and Supplementary Data 7). Using calibration curves to measure the endogenous peptide concentration (and therefore the inferred protein concentration), 217 target proteins were detected during initial assay development (in C57BL/6BR mice) and 272 target proteins were detected within the dynamic range of the assays in at least one mouse strain (in at least 4 out of 6 individual mice), across all mouse strains. Linear regression analysis on log-transformed plasma protein abundances across male and female mice and across all strains revealed near unity slope and *R*^2^ values for the majority of the comparisons (Fig. 3b and Supplementary Data 7), suggesting similar plasma protein profiles across all mouse strains and sexes. However, relatively lower slope and *R*^2^ values were observed for comparisons involving C57BL/6 mice purchased from BioReclamation (Supplementary Data 7), suggesting a somewhat different plasma protein profile in both male and female C57BL/6 mice that had been obtained from BioReclamation.

**Table 2:** Overview of mouse strains used in this study

Mouse strain	Model	Plasma protein concentration (μg μl^−1^)^a^	Source^b^
C57BL/6	Infectious diseases and diet-induced metabolic pathologies	20.1 ± 2.06	BioReclamationIVT
C57BL/6/CRL		38.6 ± 1.94	TCP (Charles River Laboratories, Wilmington, MA, USA)
C57BL/6J		42.3 ± 3.33	TCP (Jackson Laboratory, Bar Harbor, ME, USA)
129S1/SvlmJ	Transgenic and knockout model development and stem cell line production	42.0 ± 2.32	TCP
NOD/SCID/J#1303	Tumor biology and immunology	37.8 ± 3.53	TCP
BALB/cJ	Infectious diseases and immunology	39.7 ± 2.30	TCP
CD1	General multipurpose model and mutant production	45.4 ± 4.94	TCP

^a^Means ± SD are shown

^b^C57BL/6/CRL and C57BL/6J mice were purchased from the respective vendors, and housed in the Toronto Centre for Phenogenomics (TCP; Toronto, ON, CA) prior to collection of plasma

The average concentrations ± standard deviation (SD) of all proteins are listed in Supplementary Data 6 (individual replicate values are available in Supplementary Data 7). The reference plasma concentrations for these 272 proteins were determined using 6 biological replicates for each strain, which revealed unique plasma protein profiles in the individual strains. Because the genetic background can significantly impact the phenotype of knock-in and knock-out models^37^, these reference values can aid researchers in selecting suitable mouse strains for specific research applications.

### Quantitative phenotyping of mice

Genetic variability can also exist within individual inbred strains due to genetic drift, and hidden mutations which can lead to confounding results when developing models in inbred strains originating from different mouse colonies^38–40^. Additional environmental variables such as caging conditions, diet, and hydration can contribute to plasma proteome alterations.

To address potential intra-strain variability in protein abundance, MRM assays were used to compare protein abundances in C57BL/6 mice obtained from three different colonies C57BL/6BR (BioReclamation), C57BL/6J (The Jackson Laboratory), and C57BL/6/CR (Charles River Laboratories). A comparison of the protein abundances of 199 plasma proteins that were detected in all three mouse colonies revealed that the majority of proteins (~84%) had concentrations within the analytical variability of respective MRM assays (Fig. 3c, d, and Supplementary Data 6). However, 32 out of 199 proteins had varying concentrations across the three colonies of C57BL/6 mice (Fig. 3e and Supplementary Data 6), and an additional 17 proteins were not detected by MRM in at least one of the examined C57BL/6 strains. While it could not be determined whether these differences were a result of genetic drift or environmental factors, these phenotypic differences should be considered when developing mouse models using the respective strains.

The utility of these multiplexed MRM assays was further exemplified by their ease of application for deciphering immune-related differences between immunodeficient NOD/SCID mice and immunocompetent mouse strains. Inter-strain quantitative phenotyping was performed by comparing the abundance of immunoglobulins (Ig) in the plasma of immunocompetent (C57BL/6, BALB/c, 129S1, and CD1) and immuno-compromised mice (NOD/SCID). NOD/SCID mice are homozygous for the *Prkdc*^*scid*^ mutation which causes severe combined immunodeficiency, resulting in IgM, IgG1, IgG2a, IgG2b, IgG3, or IgA deficiency.

As expected, immunoglobulins were not detected in plasma from NOD/SCID mice using 17 MRM assays which targeted immunoglobulins (Fig. 3f and Supplementary Data 6), with the exception of Ig heavy chain V region MOPC 47A (which was detected in 1 out of 6 NOD/SCID mice at 2.3 fmol µL^−1^; Supplementary Data 7). A comparison of the plasma protein concentrations of immune-related proteins confirmed the severe immunoglobulin deficiencies of the NOD/SCID strain.

These results demonstrate the discriminatory power of these assays for measuring biologically relevant differences in mouse strains and confirm their applicability to deciphering of phenotypic differences between wild-type, knock-out, and/or knock-in transgenic mouse strains. It is also possible that the MRM assays described here might be applicable to other species where the target peptide contains an identical amino acid sequence (after appropriate interference testing and at least a partial validation). With respect to application of these assays in studies using human cell lines or tissues, 213 of these assays may be suitable for quantification of human proteins due to the sequence identity of the target peptides (Supplementary Data 8). These MRM assays will likely find broad application because the triple-quadrupole (QQQ)-MS instruments required for MRM are available in most core research facilities worldwide. However, MRM is often perceived as a technique that is too specialized to be applied in routine research. To make MRM more accessible to users with limited or no MS experience, our validated assays will be translated into kits for quantification of proteins.

The MRM assays described are a first step towards development of a mouse MRM atlas, a large panel of MRM assays allowing accurate and reproducible protein quantification across various mouse tissues. Our laboratory intends to use these MRM assays to determine tissue-specific protein abundances in various strains of mice (used in research), and to store these abundance values in a publicly available knowledgebase, providing a baseline to which new data can be compared to facilitate the development of novel mouse models.

## Conclusions

The reference values and assays provided in this study should prove useful to a large number of researchers, as they allow accurate and high-throughput molecular phenotyping of mouse models and, potentially, other organisms. The set of 500 precise mouse MRM assays validated according to CPTAC guidelines is the largest of its kind to be reported. Furthermore, the utility of these assays for the phenotyping of common laboratory mouse strains and their potential application to other model organisms is a promising first step towards a gold standard assay for high-throughput and specific phenotyping. Continued development of MRM assays will be vital to keep up with evolving mouse genetics, and the development of MRM assays for detectable proteins in all mouse tissues is an achievable goal.

## Methods

### Mouse plasma samples

Approval for conducting animal studies was granted by the University of Victoria Animal Care Committee (ACC). All assay development experiments were performed using plasma samples collected from C57BL/6BR mice (BioReclamationIVT; Westbury, NY, USA). Additional plasma samples from 129S1/SvlmJ, NOD/SCID/J#1303, Balb/cJ, CD1, C57BL/6/CRL, and C57BL/6 J mice (Toronto Centre for Phenogenomics, TCP; Toronto, ON, CA) were used to determine reference concentration values for the 500 target proteins (Table 2). Plasma samples were collected from three male and three female mice, fed a standard rodent chow, at 12 weeks of age (*N* = 6 for each mouse strain), with the exception of C57BL/6BR mice which were 8–10 weeks when plasma was collected.

### Chemicals and reagents

All chemicals and reagents were purchased at the highest purities available (individual vendors are listed below). Solvents used in this study were LC-MS grade and were purchased from Fisher Chemicals (Thermo Fisher Scientific, Ottawa, ON, CA).

### Sample preparation

Total plasma protein concentration in each sample was determined by the BCA assay (Thermo Fisher Scientific) according to the manufacturer’s instructions (Table 2). Individual or pooled plasma samples were processed as previously described^21^, using the procedure which is briefly summarized here. For tryptic digestion, 12.5 µL of plasma was diluted with 37.5 µL of 9 M urea, 300 mM Tris pH 8.0, and 20 mM dithiothreitol, and incubated at 23 °C for 30 min. Samples were alkylated in 40 mM iodoacetamide, incubated as above, and subsequently diluted 10 fold in 100 mM Tris pH 8.0. Trypsin was added at a protein:enzyme concentration ratio of 20:1, and samples were incubated for 18 h at 37 °C while shaking at 500 rpm. Trypsin was inactivated by addition of 1% (v/v) formic acid, and the samples were spiked with stable-isotope labelled and natural peptide mixtures specific to each experiment (Table 1). Digests were then desalted and concentrated by solid phase extraction, using OASIS HLB 96-well plates containing 30 mg sorbent per well, 30 µm particle size (Waters) according to manufacturer instructions. Eluted samples were lyophilized, and re-suspended in 0.1% aqueous formic acid. Twenty microgram of sample was injected on the LC column for each MRM experiment.

### Target protein selection

Untargeted (shotgun) LC/MS-MS was performed on an Orbitrap Fusion Tribrid coupled to an EASYnLC 1000 HPLC system via a Nanospray Flex NG source (Thermo Fisher Scientific)^21^. A 1.5-µg aliquot of trypsin digested plasma was injected onto a reversed-phase pre-column (Magic C18-AQ; 100 µm internal diameter, 2 cm length, packed with 5 µm particles, 100-Å pore size) followed by a reversed-phase nano-analytical column (Magic C-18-AQ, 75 µm internal diameter, 15 cm length, 5 µm particles, 100 Å pores) (Michrom BioResource). The solvents used for the LC gradient were 2%AcN/0.1% aqueous formic acid (A) and 90%AcN/0.1% aqueous formic acid (B). The gradient used was (%B, time in min): 3, 0; 35, 110; 45, 120; 100, 130; 100; 140, at a flow rate of 300 nL min^−1^. Electrospray ionization was performed by coupling the analytical column to a 10 µm emitter (New Objective), and the acquisition parameters were set as follows: 2500 V spray voltage, 275 °C capillary temperature, survey MS1 scan *m/z* range 200, and one microscan with an injection time of 50 ms. Data-dependent acquisition was scheduled every 3 s, with an automatic gain control of 400,000. Higher-energy collisional dissociation was used to produce peptide fragmentation, with dynamic exclusion settings set as follows: repeat count two within five seconds, exclusion duration of 10 s with a ∆ of 10 ppm. The MS2 scans used a quadrupole isolation window of 1.6 Da, a maximum injection time of 35 ms, and a stepped collision energy of 35% ± 5.

Raw data files were analyzed using Proteome Discoverer 1.4.0.228 software (Thermo Scientific). The default parameters in Proteome Discoverer were used to select the spectra for inclusion in the higher-energy collisional-dissociation peak-lists, and the peak lists were submitted to our in-house Mascot 2.4.1 server and searched against the UniProt mouse database^41^. A false-discovery rate of 1% was applied.

### Peptides

One to three peptide surrogates were selected for each protein target by our PeptidePicker software^35^. For synthesis of the SIS peptides, ^13^C/^15^N N-Fmoc l-arginine and l-lysine with 98% isotopic enrichment (Cambridge Isotope Laboratories, Andover, MA, USA) were coupled to TentaGel^TM^ R TRT resins (RAPP Polymere, Tübingen, Germany). Wang resins preloaded with non-modified N-Fmoc lysine and arginine were purchased from Matrix Innovations (Quebec City, QC, Canada). Stable isotope labelled (^13^C/^15^N) peptides and the corresponding unlabelled or natural peptides were synthesized and purified as previously described^42^. Briefly, peptides were synthesized on the appropriate resin in dimethylformamide with a 10× or 20× amino acid excess, using 40% piperidine for Fmoc deprotection, and HCTU(1 eq)/NMM (2 eq) as activator/base reagents. Peptides were cleaved from the resin and purified. The resulting purity was 88.0 ± 11.87% (mean ± SD; Supplementary Data 1). Peptides were directly infused into the MRM instrument, and MassHunter Optimizer software, version B.07.00 (Agilent Technologies, Santa Clara, CA, USA), was used to determine the dominant precursor charge state for each peptide and the five most abundant fragment ions, along with their corresponding optimal collision energies. Peptide retention times were obtained by unscheduled MRM.

For CPTAC Experiment 1, equimolar mixtures of 100–200 peptides were prepared. All other experiments were conducted with concentration-balanced peptide mixtures that reflected the dynamic ranges of the respective assays and the concentrations of the endogenous analytes in mouse plasma. For proteins where more than one surrogate peptide passed the validation criterion in CPTAC Experiment 1 and 2, a single peptide was selected for further validation (Supplementary Data 1).

### Targeted MRM

Targeted MRM was performed on an Agilent 6495 Triple Quadrupole mass spectrometer connected to a 1290 Infinity UHPLC system via a Jet Stream ESI source (Agilent Technologies), using settings which have been previously described^42^. Briefly, peptides were separated in 56-min acetonitrile (ACN) gradients at a flow rate of 0.4 mL min^−1^. The mobile phases were 0.1% formic acid in water (A) and 0.1% FA in ACN (B); the specific gradient was as follows (%B, time in min): 2, 0; 7, 2; 30, 50; 45, 53; 80, 53.5; 98, 56 with a 4-min equilibration (with 2% B) after each gradient. Targeted MS acquisitions were performed using 1-min detection windows, ≤900 ms cycle time, and ≥9 ms dwell times. For the MRM experiments, one to three transitions were used to monitor each peptide (Supplementary Data 1).

Single point quantification was performed on 500 µg C57BL/CRL plasma protein (in triplicate) using 2D-LC/MS as previously described^36^. Samples were first separated at high pH (10 mM ammonium hydroxide) using a 4.8 × 50 mm 2.5 µm C18 column (Waters) at a flow rate of 0.4 mL min^−1^. Eluting peptides were mixed online with 0.1% formic acid and trapped on a 4.6 × 50 mm 2.5 µm particle size C18 trap column (Agilent). Peptides were subsequently eluted from the trap column under acidic conditions and separated on a reversed phase analytical column (2.1 × 150 mm 1.8 µm particle size C18) (Agilent) and analyzed by MRM, as described above.

### Assay validation

A multipoint response curve was generated in a matrix consisting of digested representative mouse plasma to determine the lower limit of quantification (LLOQ) and the linear range for each of the 500 targets (CPTAC Experiment 1, Supplementary Fig. 1). Response curves were generated by spiking 12 different concentrations of SIS peptides into pooled mouse-plasma samples (Table 1). The corresponding endogenous peptides were used as normalizers to compensate for ion suppression and differences in instrument performance. For low-abundance endogenous peptides, signal intensities were increased by adding a constant amount of the respective NAT peptides to the samples (Table 1). The SIS:NAT signal intensity ratios were plotted against the concentrations of the spiked-in SIS peptides to generate the response curves. In parallel, the concentrations of the endogenous peptides were determined using the corresponding SIS peptide (*c* = 200 fmol on LC column/injection) as a normalizer.

The lower limit of quantification (LLOQ) of the assay was defined as the spiked-in SIS concentration where the intra-assay coefficient of variation (CoV) was less than 20%, adjusted (if possible) to the concentration of the endogenous analyte. The LLOQ of the assay is different from the LLOQ of the peptide—the latter corresponds to the lowest spiked-in SIS concentration with CoV of <20%. Using the assay LLOQ instead of the peptide LLOQ for assay development ensured that the endogenous analyte concentration was approximately in the middle of the dynamic range, thus improving the analytical performance of the assay. All 500 response curves were linear over a concentration range of ≥1000x the LLOQ, and were validated according to the CPTAC guidelines.

To assess the repeatability of the assays, pooled mouse plasma samples were prepared and analyzed in five independent MRM runs (CPTAC Experiment 2, Supplementary Fig. 1). Three aliquots of the representative digested plasma matrix were individually spiked with low (2.5× and 5× assay LLOQ), medium (50× assay LLOQ) and high (500× assay LLOQ) concentrations of SIS peptides and a constant concentration of the corresponding NAT peptides (Table 1). Samples were independently spiked and were subsequently analyzed by MRM on five different days. For each SIS peptide concentration, the intra-assay variability was defined as the average of the CoV values determined on each of the five days. Inter-assay variability was also determined for each SIS peptide concentration by averaging the CoV values determined for each of the 5 process replicates (individual injections) across all five days. Finally, the total variability was calculated as the square root of the sum of squared intra-assay and inter-assay CoVs.

For CPTAC Experiment 1, five transitions for each peptide were monitored by MRM. Extracted ion chromatograms for each peptide were analyzed using SkylineDaily^43^. The three most intense interference-free transitions were selected for the subsequent validation steps, with the most intense transition serving as a quantifier, and the other two serving as qualifiers (transitions used for quantification are listed in Supplementary Data 1). SIS/NAT (CPTAC Experiments 1, 2, and 4), NAT/SIS (CPTAC Experiment 3), or endogenous/SIS (CPTAC Experiment 5) signal ratios were exported from Skyline, and used for subsequent analysis. Data were analyzed using Qualis-SIS software^44^ and/or GraphPad Prism, version 7.03 (La Jolla, CA, USA). In CPTAC Experiment 1, the response curves were generated by linear regression over a minimum of three consecutive concentration levels (20,000 to 0.16 fmol on column; dilution pattern: 1:10:10:5:2:2:2:2:2:2:2:2) using 1(*x*^2^)^−1^ weighting. Curves were required to have a precision and accuracy of 20% or less, for each level^44^(otherwise the assay was not validated in subsequent CPTAC experiments). The experimental designs of all validation experiments are summarized in Table 1.

### Target protein quantification in laboratory mouse strains

Digested plasma samples from individual mice (Table 2) were spiked with SIS peptides (100× assay LLOQ; Table 1). In parallel, a calibration curve was prepared by spiking serial dilutions of NAT peptides (1000× to 1× assay LLOQ; dilution pattern: 1:5:2.5:4:2:2:2:2) and the corresponding SIS peptides (100× assay LLOQ) into digested BSA (Sigma Aldrich, Oakville, ON, CA) as the matrix. Additionally, quality control (QC) samples A–C were prepared in triplicate by spiking NAT peptides (A: 300×, B: 30×, and C: 4× LLOQ) and the corresponding SIS peptides (100× assay LLOQ) into the BSA matrix. All samples were then processed and analyzed by MRM as described above.

Molecular weights of target proteins were computed using the ExPASy pI/Mw tool and the UniProt accession numbers^41^, and represent the average molecular weights of the mature proteins without signal sequences (Supplementary Data 6). For proteins with multiple cleavage products, the average molecular weights of the full-length mature proteins were selected; all additional cleavage products that could potentially be quantified by the respective assays are listed in Supplementary Data 6, and the MRM assay annotations are highlighted in bold. For proteins with cleavage products that do not overlap, the average molecular weight of the product containing the surrogate peptide was selected, and the assay annotation was changed accordingly (Supplementary Data 6; yellow-shaded cells). Finally, the average molecular weight of the full-length protein sequence was selected for IgG chain fragments (P01786, P03987, P01864, P01867, P01869, P01878, P01630, P01631, P01636, P03976, P01750, P04945, P01643, P01872, P01635, P01837, and P01674; Supplementary Data 6; blue-shaded cells). The surrogate peptide VAPEEHPVLLTEAPLNPK matches two proteins, P60710 and P63260 (Actin cytoplasmic 1 and 2, respectively), so the molecular weights of both target proteins are listed (Supplementary Data 6).

Concentrations of the endogenous analytes were calculated from the endogenous/SIS signal ratios of the respective quantifiers using the standard curve. The calculated concentrations were then adjusted for the purity and the measured concentration of the SIS normalizer (as determined by the capillary zone electrophoresis, and the amino acid analysis, respectively^42^) (Supplementary Data 1). Protein concentrations in mouse plasma were back calculated from the amount of endogenous peptide measured from the standard curves, and after taking the protein (and/or isoform) molecular weight into consideration, for reporting protein concentration in µg mL^−1^.

### Statistical analysis

Mouse plasma from all strains were assigned numbers and processed through to the end of data analysis until protein quantification was complete, without knowledge of corresponding mouse strain. Figure 3e: Individual concentrations of endogenous analytes measured in plasma of C57BL/6BR, C57BL/6/CRL, and C57BL/6J mice were first normalized to the median concentrations of the respective analytes in plasma of C57BL/6BR mice. Subsequently, data were analyzed by two-way ANOVA with Tukey’s correction for multiple comparisons (GraphPad Prism v.6.0). Comparisons were considered significant if *p* < 0.05.

### Data availability

The data reported in this study are summarized in the manuscript and its Supporting Information files. Validation data and assay parameters are available from the Panorama portal (https://panoramaweb.org/), and will be also uploaded to the CPTAC assay portal (https://assays.cancer.gov/). Raw data pertaining to the CPTAC experiments were also deposited in PeptideAtlas (http://www.peptideatlas.org/). Proteomic datasets for experiments 1 and 2 can be accessed using the dataset identifier PASS01163. Proteomic datasets for experiments 3 through 5 can be accessed using the dataset identifier PASS01200.

## Electronic supplementary material

Supplementary Information

Description of Additional Supplementary Files

Supplementary Data 1

Supplementary Data 2

Supplementary Data 3

Supplementary Data 4

Supplementary Data 5

Supplementary Data 6

Supplementary Data 7

Supplementary Data 8
