# Supplementary material for: Molecular phenotyping of laboratory mouse strains using 500 multiple reaction monitoring mass spectrometry plasma assays
Source: Commun Biol. 2018 Jun 27;1:78. doi: 10.1038/s42003-018-0087-6 (PMC6123701; doi:10.1038/s42003-018-0087-6)
Supplement: Supplementary file 1 — Supplementary Information [file 42003_2018_87_MOESM1_ESM.pdf]

Supplementary Figure 1. CPTAC Schematic Representation of the Assay Characterization Guidance Document

(reprinted with permission from https://proteomics.cancer.gov/sites/default/files/CPTAC%20Schematic%20Representation%20of%20the%20Assay%20Characterization%20Guidance%20Document.pdf).

Assay Development Working Group - Experiments for Assay Characterization

Experiment 1: *Response Curve*

- Development of multipoint response curve (1 blank and a minimum of 6 concentration points).
- Samples prepared in digested matrix background (i.e. plasma, tissue, cells, etc).
- Used for the determination of LOD, LLOQ and linearity.
- Multiple replicates analyzed.

Experiment 2: *Mini-Validation of Repeatability*

- Examines intra- and inter-assay variability.
- Uses the LLOQ from Experiment 1 from which 3 concentrations (Low, Medium and High) are used to assess repeatability.
- 3 replicates processed and measured on 5 different days.

Experiment 3: *Selectivity*

- Examines the response of a peptide in six different biological replicates of the matrix.
- Replicates analyzed with no spike and ½ the Medium and Medium concentrations defined in Experiment 2.

Experiment 4: *Stability*

- Examines the stability of a peptide spiked into a background matrix
- Stability assessed based on peak area variability following:
  - different storage conditions (4C and -70C) over time.
  - freeze-thaw cycles
- Variability compared to data collected from Experiment 2.

Experiment 5: *Reproducible Detection of Endogenous Analyte*

- Representative sample containing endogenous analyte is digested 5 times on each of 5 days.
- Examines intra- and inter-assay variability of the entire assay workflow, including digestion.

Experiment 1: ***Response Curve***

KEY POINTS

- Development of multipoint response curve (1 blank and a minimum of 6 concentration points).
- Samples prepared in digested matrix background (i.e. plasma, tissue, cells, etc).
- Used for the determination of LOD, LLOQ and linearity.
- Multiple replicates analyzed.

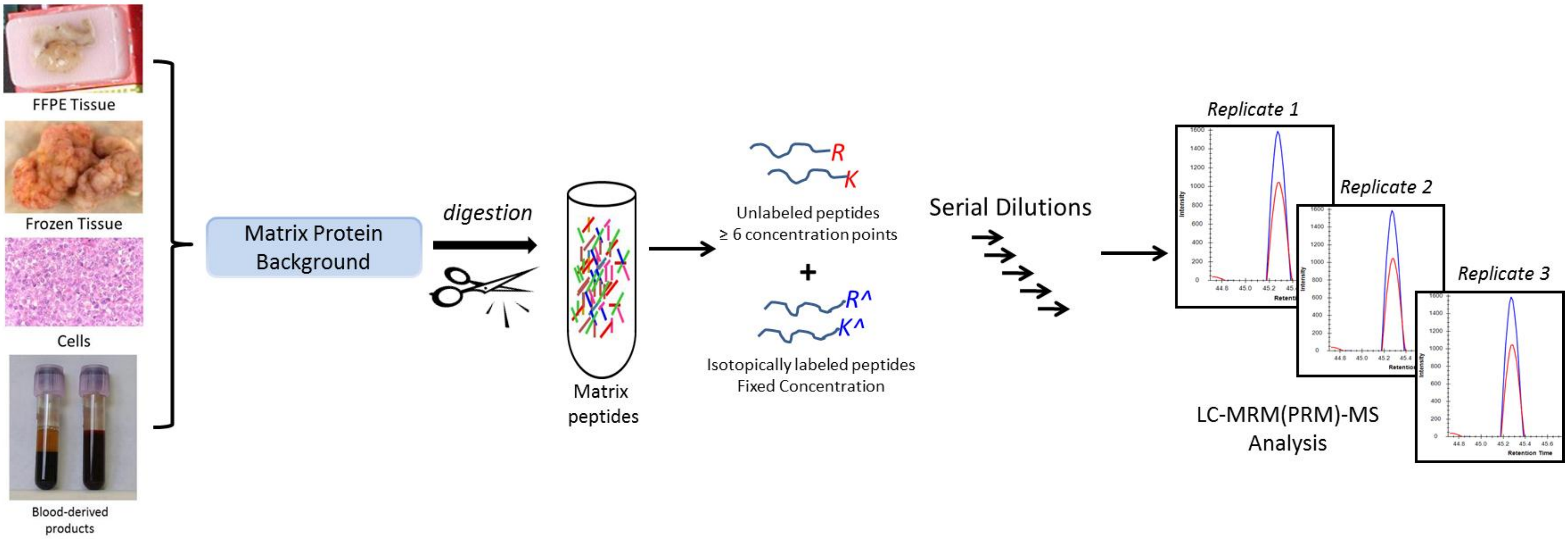

Experiment 2: ***Mini-Validation of Repeatability***

KEY POINTS

- Examines intra- and inter-assay variability.
- Uses the LLOQ from Experiment 1 from which 3 concentrations (Low, Medium and High) are used to assess repeatability.
- 3 replicates processed and measured on 5 different days.

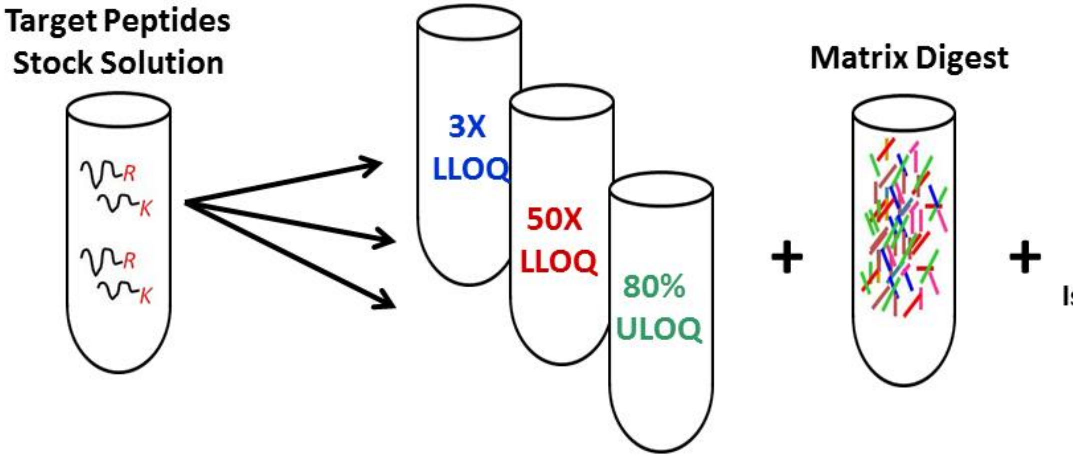

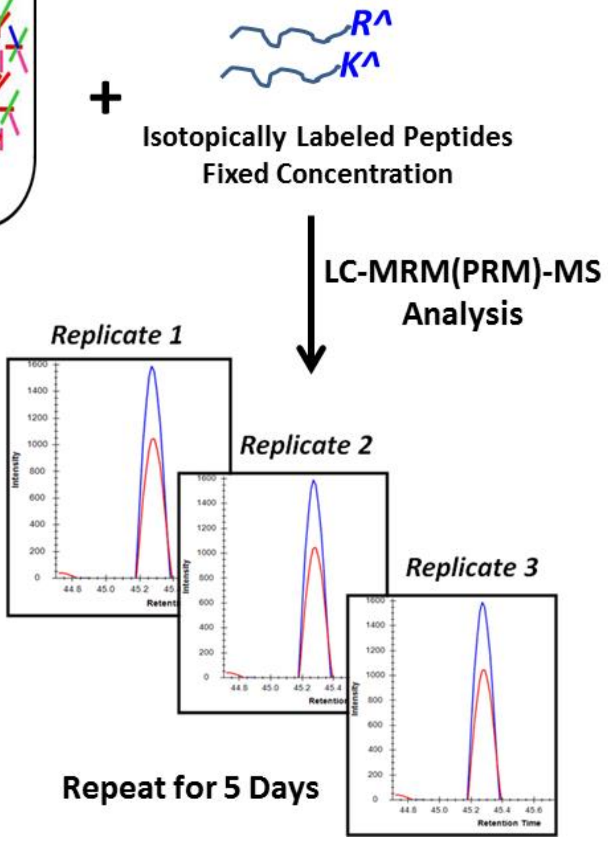

		**Replicate**			
**Day**	**1**	**2**	**3**		
**1**	2156	1989	2095		4.1%
**2**	2066	1653	2103		12.9%
**3**	1509	1755	1536		8.4%
**4**	1862	2132	2454		13.8%
**5**	1617	1515	1724		6.5%
				**Ave**	9.1%
	15.1%	13.8%	18.1%	15.7%	
				**Total error**	18.1%

Experiment 3: ***Selectivity***

KEY POINTS

- Examines the response of a peptide in six different biological replicates of the matrix.
- Replicates analyzed with no spike and ½ the Medium and Medium concentrations defined in Experiment 2.

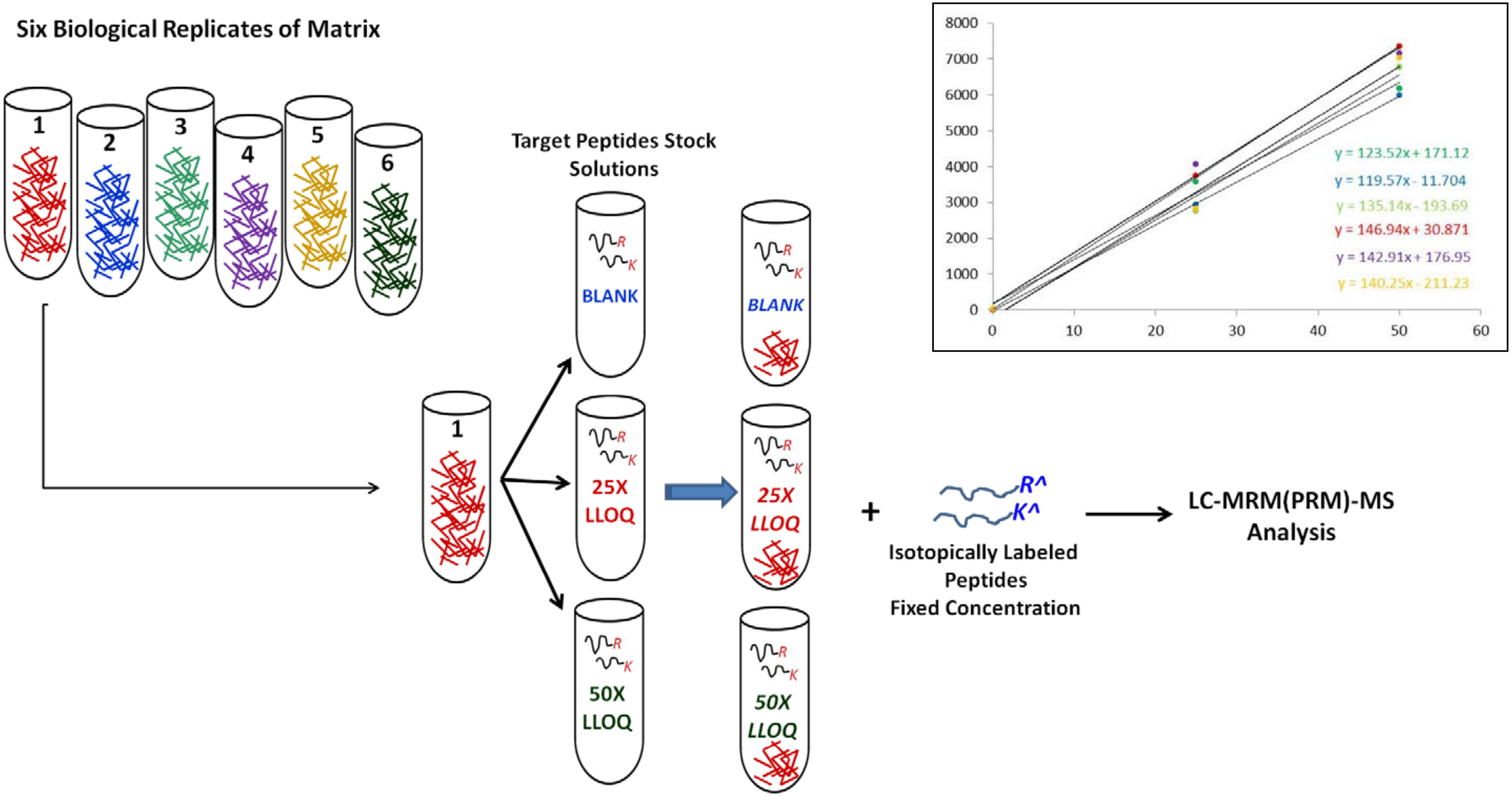

Experiment 4: ***Stability***

KEY POINTS

- Examines the stability of a peptide spiked into a background matrix
- Stability assessed based on peak area variability following:
  - different storage conditions (4C and -70C) over time.
  - freeze-thaw cycles
- Variability compared to data collected from Experiment 2.

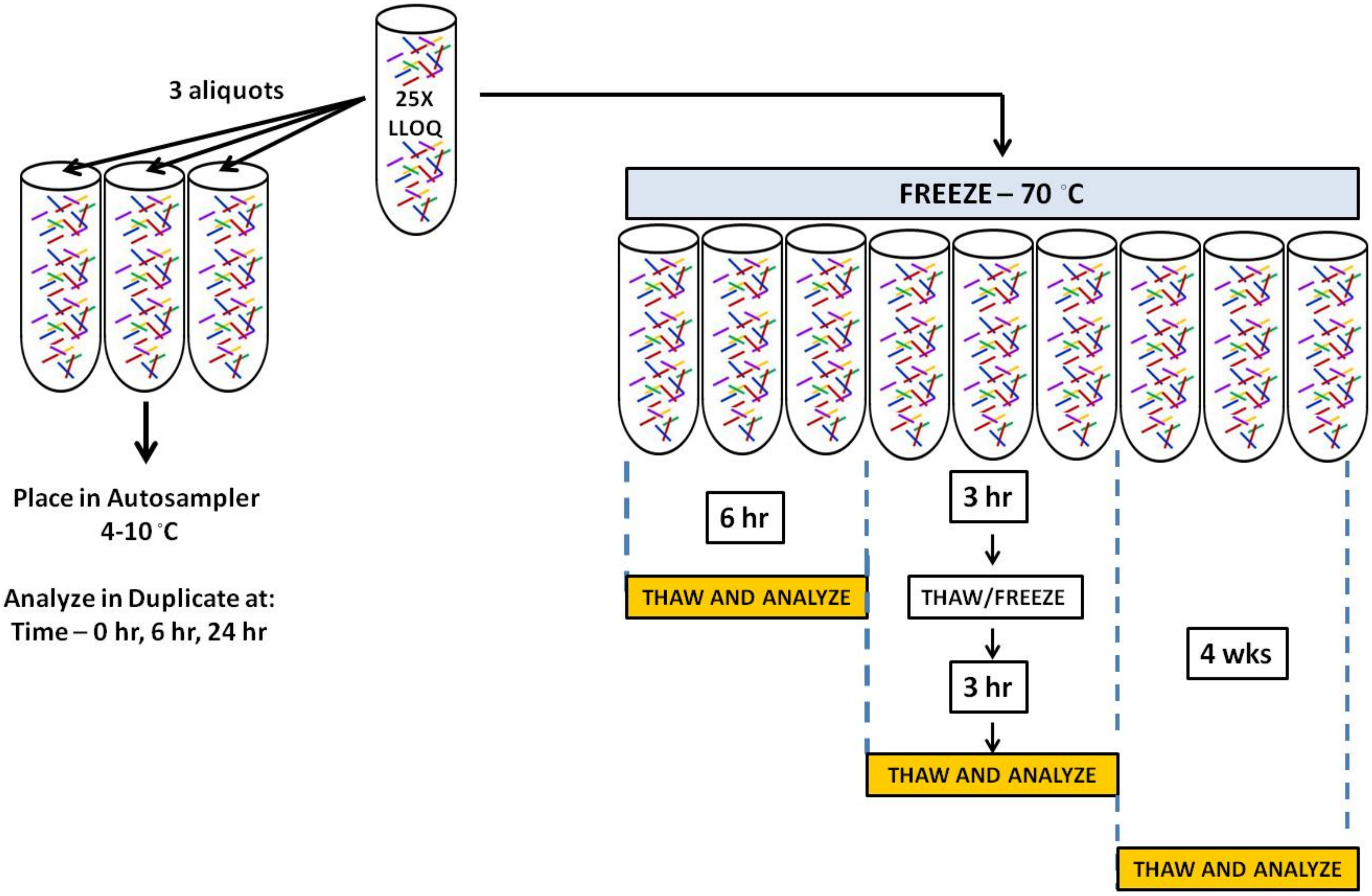

Experiment 5: ***Reproducible Detection of Endogenous Analyte***

KEY POINTS

- Representative sample containing endogenous analyte is digested 5 times on each of 5 days.
- Examines intra- and inter-assay variability of the entire assay workflow, including digestion.

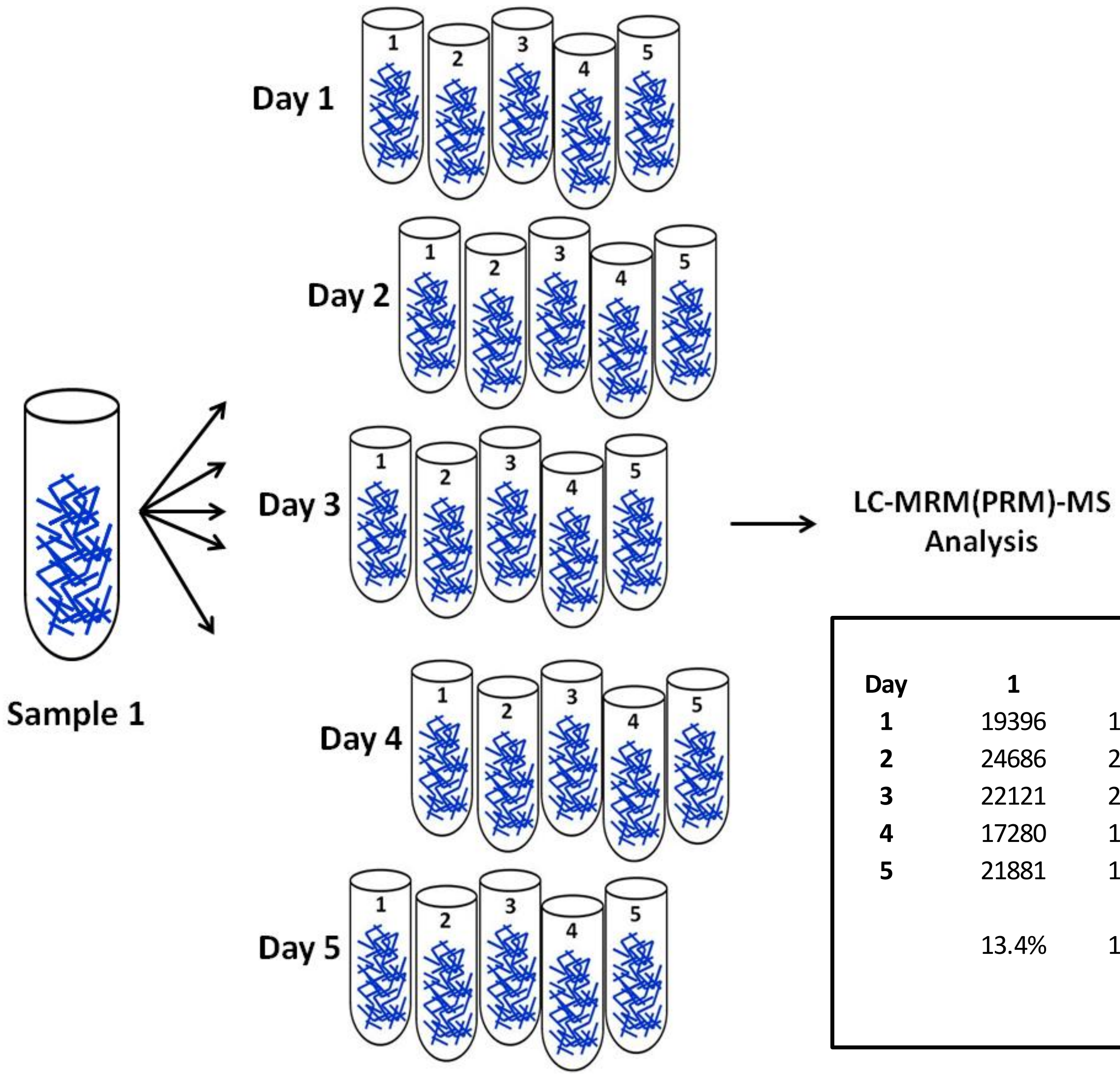

	Replicate						
**Day**	**1**	**2**	**3**	**4**	**5**		
**1**	19396	17334	17325	15488	16573		8.3%
**2**	24686	21386	21000	23260	22226		6.6%
**3**	22121	26532	22050	20064	26730		12.7%
**4**	17280	18572	20295	14404	21384		14.8%
**5**	21881	19859	21450	16840	22113		10.7%
						**Ave**	10.6%
	13.4%	17.2%	9.0%	20.1%	16.5%	13.2%	
						**Total error**	17.0%
