# Supplementary material for: Molecular phenotyping of laboratory mouse strains using 500 multiple reaction monitoring mass spectrometry plasma assays
Source: Commun Biol. 2018 Jun 27;1:78. doi: 10.1038/s42003-018-0087-6 (PMC6123701; doi:10.1038/s42003-018-0087-6)
Supplement: Supplementary file 2 — Description of Additional Supplementary Files [file 42003_2018_87_MOESM2_ESM.docx]

**Description of Additional Supplementary Files**

File Name: Supplementary Data 1

Description: **Optimized parameters for MRM mouse plasma assays and purity-adjusted SIS peptide concentrations.**

AAA – amino acid analysis, CZE – capillary zone electrophoresis

File Name: Supplementary Data 2

Description:  **Total variability and lower limit of quantification of MRM mouse plasma assays (CPTAC Experiment 2).**

Blue- and yellow-shaded cells represent total variability at the 2.5x and 5x assay LLOQ , respectively.

File Name: Supplementary Data 3

Description: **Stability of MRM mouse plasma assays (CPTAC Experiment 4).**

Table summarizes variability and technical variability of each assay at six experimental time points spanning 4 weeks.

T – freeze-thaw, wks – weeks.

File Name: Supplementary Data 4

Description: **Reproducibility of target endogenous analyte detection in pooled plasma from (CPTAC Experiment 5).**

ND –endogenous analyte not detected

File Name: Supplementary Data 5

Description: **Selectivity of MRM mouse plasma assays (CPTAC Experiment 3)**

Table summarizes the slopes of the response curves of each assay in the plasma of individual C57BL/6BR mice (M436 – M441).

ND –endogenous analyte not detected

File Name: Supplementary Data 6

Description: **Reference concentrations of 272 target proteins in 5 mouse strains.**

Concentrations of 500 target analytes were measured by the CPTAC-validated MRM assays in plasma from C57BL/6 mice (C57BL/6BR from BioreclamationIVT, C57BL/6/CRL originating in the Charles River Laboratories, and C57BL/6J mice originating in the Jacksons Laboratories), CD1, 129S1/SvlmJ, NOD/SCID/J#1303, and Balb/cJ mice. Reference values are reported only for analytes detected within the dynamic range of the respective assays in plasma of at least 4 mice per mouse strain (N=272). For the protein Q19LI2, reference values for males and females are listed separately due to distinct sex-specific differences in protein abundance. ULOQ – upper limit of quantification. Molecular weight (MW) of target mature proteins (without signal sequences) used for calculation of protein concentration (in ng/ml) is shown. For proteins with multiple cleaving products, the average molecular weights of the full-length mature protein are listed; all additional cleaving products containing the surrogate peptide are highlighted in bold. For proteins with cleaving products that do not overlap, the average molecular weight of the product containing the surrogate peptide is shown (yellow-shaded cells). Finally, the average molecular weight of the full-length protein sequences of IgG chain fragments is listed (blue-shaded cells). Surrogate peptide VAPEEHPVLLTEAPLNPK matches two proteins, P60710 and P63260 (Actin cytoplasmic 1 and 2, respectively); molecular weights of both proteins are listed. Grey-shaded cells represent statistically significant comparisons (p<0.05) as determined by two-way ANOVA with Tukey’s correction for multiple comparisons; a and b stand for statistically significant comparisons between the indicated vendors. Green-shaded cell highlight lower immunoglobulin (Ig) abundance in immunocompromised NOD/SCID/J#1303 mice.

File Name: Supplementary Data 7

Description: **Concentrations of 500 target analytes in 5 mouse strains (raw data).**

M – male, F – female, ND – analyte not detected or detected below the lower limit of quantification of the assay

ULOQ – analyte detected above the upper limit of quantification (ULOQ).

File Name: Supplementary Data 8

Description: **Mouse proteotypic peptides that are identical to those for human proteins.**
